# Hereditary Syndromes Associated with Pancreatic and Lung Neuroendocrine Tumors

**DOI:** 10.3390/cancers16112075

**Published:** 2024-05-30

**Authors:** Nektaria Papadopoulou-Marketou, Marina Tsoli, Eleftherios Chatzellis, Krystallenia I. Alexandraki, Gregory Kaltsas

**Affiliations:** 1 Neuroendocrine Tumor Unit, EURACAN 4 and ENETS Centre of Excellence, 1st Department of Propaedeutic Internal Medicine, Laiko General Hospital, National and Kapodistrian University of Athens, 11527 Athens, Greece; martso.mt@gmail.com (M.T.); gregory.kaltsas@gmail.com (G.K.); 2 251 Air Force General Hospital, 11525 Athens, Greece; lefterchatz@gmail.com; 3 2nd Department of Surgery, Aretaieio Hospital Athens, Medical School, 11528 Athens, Greece; alexandrakik@gmail.com

**Keywords:** hereditary syndromes, pancreatic neuroendocrine tumors, lung neuroendocrine tumors, MEN1

## Abstract

**Simple Summary:**

Pancreatic neuroendocrine tumors (PanNETs) and lung NETs (LNETs) have garnered increased attention in recent years due to their diverse clinical manifestations and challenging management. Although the vast majority of both PanNETs and LNETs are sporadic, they can also occur in the context of inherited syndromes that necessitate a high index of suspicion as the course of the disease is different and there are additional clinical implications for other family members. Our systematic review encompasses studies conducted over the past decade, focusing on the incidence, diagnosis, and treatment of PanNETs and/or LNETs encountered in hereditary syndromes such as Multiple Endocrine Neoplasia type 1 (MEN1), MEN4, von Hippel–Lindau (VHL), Tuberous Sclerosis Complex (TSC), and Neurofibromatosis type 1 (NF1). We discuss the importance of early detection and tailored management strategies, emphasizing the need for multidisciplinary collaboration among relevant healthcare professionals to optimize patients’ outcomes. Moreover, we highlight the importance of active surveillance strategies for early tumor detection in high-risk individuals and the evolving landscape of personalized medicine for targeted intervention.

**Abstract:**

Pancreatic neuroendocrine tumors (PanNETs) and lung NETs (LNETs) represent a rare but clinically significant subgroup of neoplasms. While the majority is sporadic, approximately 17% of PanNETs and a subset of LNETs develop in the context of monogenic familial tumor syndromes, especially multiple endocrine neoplasia type 1 (MEN1) syndrome. Other inherited syndromes associated with PanNETs include MEN4, von Hippel–Lindau (VHL) syndrome, neurofibromatosis type 1 (NF1), and tuberous sclerosis complex (TSC). These syndromes are highly penetrant and their clinical manifestations may vary even among members of the same family. They are attributed to genetic mutations involving key molecular pathways regulating cell growth, differentiation, and angiogenesis. Pancreatic NETs in hereditary syndromes are often multiple, develop at a younger age compared to sporadic tumors, and are associated with endocrine and nonendocrine tumors derived from multiple organs. Lung NETs are not as common as PanNETs and are mostly encountered in MEN1 syndrome and include typical and atypical lung carcinoids. Early detection of PanNETs and LNETs related to inherited syndromes is crucial, and specific follow-up protocols need to be employed to optimize diagnosis and management. Genetic screening is recommended in childhood, and diagnostic screening starts often in adolescence, even in asymptomatic mutation carriers. Optimal management and therapeutic decisions should be made in the context of a multidisciplinary team in specialized centers, whereas specific biomarkers aiming to identify patients denoted to follow a more aggressive course need to be developed.

## 1. Introduction

**Figure 1:**
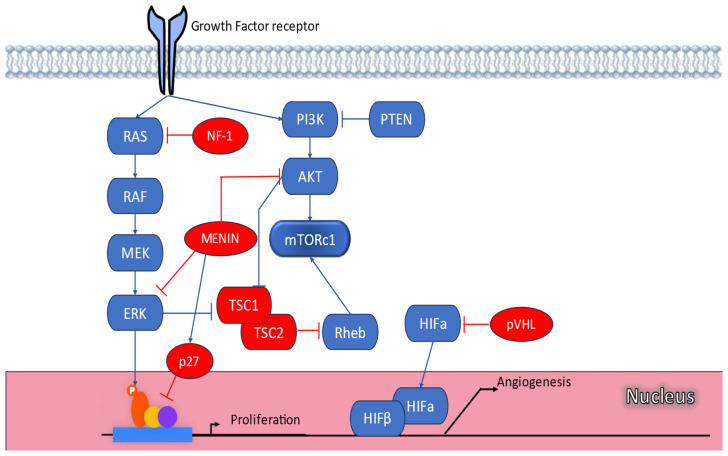
Molecular mechanisms and pathways that have been implicated with tumorigenesis associated with hereditary syndromes associated with PanNETs and LNETs. (P13K: Phosphatidylinositol-3-kinases, PTEN: phosphatase and tensin homolog, AKT: protein kinase B, mTORc1: mechanistic target of rapamycin complex 1, RheB: RAS homolog enriched in brain, RAS: rat sarcoma virus, NF-1: neurofibromatosis type 1, RAF: proto-oncogene, MEK: mitogen activated protein kinase, ERK: extracellular signal-regulated kinase, TSC1: tuberous sclerosis 1 (hamartin), TSC2: tuberous sclerosis complex2, P27: (Kip1), member of a family of CDK inhibitors (CDIs), HIFα: hypoxia inducible factor α-subunit, HIFβ: hypoxia inducible factor β-subunit, pVHL: von Hippel–Lindau tumor protein).

Pancreatic neuroendocrine tumors (PanNETs) are rare neoplasms that constitute approximately 1–2% of all pancreatic cancers. The incidence of PanNETs has increased over the last decades, most recently reported as 0.48 cases per 100,000 per year [1]. The vast majority of PanNETs are sporadic, but approximately 17% are associated with an inherited syndrome, the most common being Multiple Endocrine Neoplasia type 1 (MEN1), von Hippel–Lindau (VHL) syndromes, Neurofibromatosis type 1 (NF1), and Tuberous Sclerosis Complex (TSC) [2,3]. Recently, a novel MEN syndrome (MEN4) with clinical features that overlap with other MEN syndromes has been described, and a few cases with PanNETs have been reported [4,5]. These hereditary syndromes are related to distinct molecular alterations that lead to the development of tumors in multiple endocrine and nonendocrine tissues (Figure 1, Table 1) [2]. Hereditary PanNETs commonly develop at an earlier age than sporadic ones, may precede or follow other clinical manifestations of the syndrome, and determine patients’ prognosis [6]. MEN1-associated PanNETs are often multifocal and have a relatively more indolent course compared to sporadic PanNETs [7,8]. Although the majority is not related to the secretion of biologically active substances leading to distinct clinical syndromes (nonfunctioning PanNETs), approximately 30% are functioning. The most frequent functioning PanNETs in MEN1 and TSC are insulinomas, while in VHL, PanNETs are almost exclusively nonfunctioning [9]. 

Lung NETs (LNETs) comprise a heterogenous group of tumors, ranging from well-differentiated bronchial typical (TC) or atypical (AC) carcinoids to poorly differentiated and highly malignant small-cell lung cancer (SCLC) and large-cell neuroendocrine carcinoma (LCNEC) [10]. Poorly differentiated LNETs are not encountered as distinct entities in these syndromes, and the term LNETs will be solely referred to well-differentiated LNETs. LNETs are usually slow-growing neuroendocrine epithelial malignancies, with an annual incidence ranging from 0.2 to 2/100,000 persons [10]. Histologically confirmed LNETs have been reported in 5% of MEN1 patients, while radiologically suspicious lung lesions may be observed in approximately 23% of cases [7,11,12]. MEN1-associated LNETs typically follow an indolent course and display a good prognosis [12,13]. In addition, thymic NETs (TNETs) may also develop in such patients, albeit they comprise a distinct entity from LNETs and will be described briefly.

The aim of this study was to review the current knowledge on inherited syndromes associated with PanNETs and LNETs and to discuss the controversies regarding the proper management that ensures the optimal outcome of patients with hereditary NETs.

## 2. Methods

This systematic review aimed to summarize the latest literature focusing on pancreatic neuroendocrine tumors, lung carcinoids, and associated hereditary syndromes. The review adhered to the Preferred Reporting Items for Systematic Reviews and Metanalyses (PRISMA) 2020 and incorporated the Quality Assessment of Diagnostic Accuracy Studies (QUADAS) guidelines to ensure transparency and rigor in the review process. 

A comprehensive search strategy was developed to identify relevant studies across electronic databases, including PubMed, EMBASE, and the Cochrane Database of Systematic Reviews. The search strategy incorporated Medical Subject Headings (MeSH) terms and keywords related to the target conditions and included Boolean operators to combine search terms effectively.

The study selection process included full-text assessment of relevant publications to determine their relevance based on predetermined inclusion and exclusion criteria.

Inclusion criteria considered studies (epidemiological studies, observational/cohort studies, retrospective/prospective studies, systematic reviews, narrative reviews, and consensus articles) written in English, published between January 2010 and 29 February 2024, focusing on pancreatic neuro-endocrine tumors (PanNETs or pNets or pNens), lung carcinoids (LNETs, Typical or Atypical Lung carcinoids), Thymic NETs or carcinoids, and associated hereditary syndromes (MEN1 or MEN4, VHL, TSC, NF1).

The exclusion criteria included animal studies and review articles not meeting inclusion criteria. 

The data from included studies were extracted using a standardized data extraction form, capturing study characteristics, methodology, and key findings. Initial study selection was conducted by assessing titles and abstracts to establish eligibility. Studies that did not fulfill the specified criteria were excluded at this point. A second screening process was conducted on the remaining research, during which full articles were read. The studies’ quality was evaluated using the QUADAS tool. Findings from included studies were synthesized descriptively to provide a comprehensive overview of the literature. A total of 1469 records were identified, screened, and assessed for eligibility, and 118 manuscripts were included in the review. 

## 3. Multiple Endocrine Neoplasia Type 1 (MEN1)

MEN1 is a rare autosomal dominant disorder with an overall estimated prevalence of 3–10/100,000 inhabitants [14,15]. MEN1 displays a high penetrance, and more than 95% of the mutation carriers develop disease-associated manifestations before the age of 50 years [7,16]. The cardinal clinical manifestations of MEN1 are primary hyperparathyroidism (pHPT), duodenopancreatic neuroendocrine tumors (dpNETs), and anterior pituitary adenomas (PitNETs), with a lifetime risk of clinical presentation of 95%, 80%, and 50%, respectively [7]. Thymic, lung, and gastric NETs and adrenal adenomas are other endocrine tumors encountered in MEN1. Cutaneous lesions such as collagenomas and angiofibromas, lipomas, and meningiomas also develop in carriers of the MEN1 gene, while female patients may have an increased risk of breast cancer [7]. Among all the manifestations, TNETs and PanNETs contribute significantly to the morbidity and mortality of MEN1, leading to a decreased life expectancy in MEN1 patients compared to the general population [17]. 

**Figure 2:**
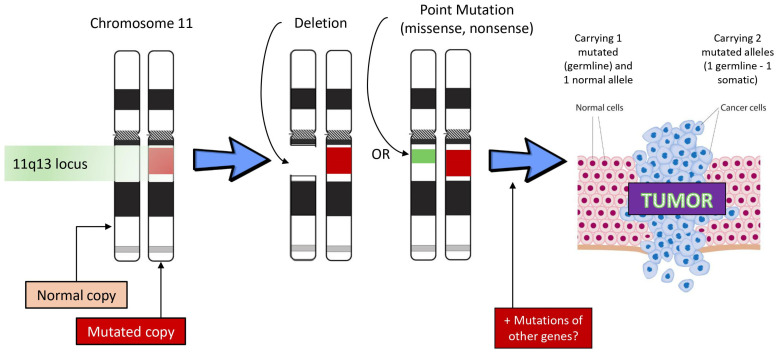
Steps in oncogenic process in mutation carriers of the MEN1 gene. The mutant menin gene is inherited from an affected progenitor or arises de novo (10%) and is present in all cells in all tissues. The normal allele produces sufficient amounts of the menin protein so that its tumor suppressor effect is present in all apparent “normal” cells in many tissues. However, in cells of some specific tissues related to the clinical phenotype, a somatic deletion or mutation of the normal allele causes complete loss of the function of the unaffected menin gene (no functional gene product or truncated protein), (+/− other gene mutations), leading to complete loss of menin production in these particular tissues and subsequent tumor formation.

MEN1 is caused by germline mutations in the MEN1 gene located on chromosome 11q13 and consisting of 10 exons [4]. The gene encodes menin, a nuclear protein of 610 amino acids with tumor suppressor properties, which plays a significant role in transcription regulation, genome stability, and cell growth control [18] (Figure 2). Its loss results in uncontrolled cell proliferation, leading to tumor development in endocrine and nonendocrine tissues [17,19]. The genetic alterations in the MEN1 gene include large nucleotide deletions and truncating, missense, or splicing point mutations [20,21]. Thus far, no mutational hotspot has been recognized and no genotype–phenotype relationship has been established. However, a recently published study providing an overview of MEN1 variants present in French families with MEN1 reported a genotype–phenotype association for large rearrangements in the MEN1 gene that were associated with earlier onset of disease compared to truncating or missense variants [22].

The diagnosis of MEN1 may be established according to three categories of criteria. The genetic diagnosis is based on the identification of a pathogenic germline mutation in the MEN1 gene in an individual who does not have clinical, biochemical, or radiological manifestations of MEN1 (i.e., a mutant gene carrier). The familial diagnosis is established when a patient with one MEN1-associated tumor has a first-degree relative with MEN1 and the clinical diagnosis is made if a patient has two or more cardinal MEN1-associated endocrine tumors [16]. 

Genetic testing is suggested in patients with clinical or familial diagnosis, in first-degree relatives of MEN1 patients, starting at the age of 5 years, as well as in patients with suspicious or atypical manifestations, including multiglandular parathyroid disease, multiple PanNETs, gastrinoma, development of pHPT before the age of 30 years, and presence of two or more MEN1-associated tumors not meeting the clinical criteria for making the diagnosis [16]. At present, a pathogenic variant of MEN1 is identified in approximately 90% of familial and 44% of sporadic PanNETs, respectively [2,23,24]. A possible explanation for the negative genetic test in clinically diagnosed patients is the presence of unidentified pathogenic variants in noncoding or regulatory regions that are not analyzed during the routine genetic testing. In addition, there are recently identified hereditary syndromes with an MEN1-like phenotype attributed to mutations in other genes, while there is a possibility of sporadic development of two MEN1-related tumors [2,4,5]. A recent study using the DMSG database reported that mutation-negative patients develop MEN1 manifestations at a later age and have a life expectancy similar to the general population [25]. The majority of them do not develop a third cardinal manifestation of MEN1 and mostly present with the combination of pHPT and pitNET. Similarly, a different clinical course for genotype-negative compared to genotype-positive MEN1 index cases and pHPT was reported [26]. Hence, a tailored surveillance protocol and a more limited follow-up may be recommended in patients with negative genetic analysis [7,26].

## 4. Pancreatic NETs in MEN1

PanNETs develop in up to 80% of MEN1 patients with a cumulative incidence that increases with age [27]. Data from the French Groupe d’Etude des Tumeurs Endocrines (GTE) and the DutchMEN Study Group (DMSG) showed that more than 80% of patients with MEN1 develop a duodeno-pancreatic NET by the age of 80 years [2,28,29]. MEN1-related PanNETs are often multiple, nonfunctioning (NF-PanNETs) and may be diagnosed early in the second decade of life [16,30]. The penetrance of NF-PanNETs is high, with a cumulative probability of developing an NF-PanNET of 12% and 16.1% at ages 18 and 21 years, respectively [29]. Functioning PanNETs in patients with MEN1 include insulinomas (10%), rarely gastrinomas, glucagonomas (<1%), somatostatinomas (1%), and vasoactive intestinal peptide-secreting tumors (VIPomas) (<1%) [9]. The vast majority of MEN1-associated gastrinomas are located in the duodenum [9]. PanNETs rarely develop in young MEN1 patients, with the youngest patient having been diagnosed at the age of 8 years [6,22,31,32]. Following the systematic use of endoscopic ultrasound (EUS) in young MEN1 patients, the prevalence of clinically occult (nonfunctioning) PanNETs was reported in up to 42% in pediatric cases [33]. MEN1-related PanNETs are smaller than sporadic ones in size, and usually occur on a diffuse micro-adenomatosis background [34]. 

Insulinomas occur with an incidence of 10–15% in patients with MEN1 and may develop at a very young age [22,32]. A recently published large international multicenter study reported that 16% of patients with insulinomas were younger than 21 years at diagnosis [35]. Insulinomas were the most frequent PanNETs in the juvenile (<21 years old) MEN1 GTE cohort and can be diagnosed even at five years of age [32]. Insulinomas frequently present as a single lesion in the context of multiple concomitant NF-PanNETs, although surgical series have reported multiple insulinomas in 8–40% of cases [7]. Symptoms may be neuroglycopenic (irritability, confusion, abnormal behavior, visual disturbances, cognitive deficits, coma) and/or adrenergic, including tachycardia, sweating, hunger, and tremor [9]. The average time between the onset of symptoms and final biochemical diagnosis is at least 2 years but, in some cases, may extend up to 5 years [9].

Gastrinomas causing the Zollinger–Ellison syndrome (ZES) usually occur later in life at a mean age 44 years (on average, ten years earlier than sporadic gastrinomas) and usually appear as small lesions arising in the duodenum (>80%) [36,37]. It has been reported that 20–60% of patients with ZES are diagnosed with MEN1 [36,38]. In the juvenile GTE cohort, 2% of patients were diagnosed with a gastrinoma [32]. Gastrinomas are rare in children with MEN1, but when present, they can be aggressive and present with lymph node and liver metastases in 34–85% and 6–16% of cases, respectively, at the time of diagnosis [22,32]. The presence of multiple, recurrent, refractory ulcers or ulcers on atypical locations, severe gastroesophageal reflux disease (GERD), and/or diarrhea that is responsive to proton pump inhibitors (PPI) should raise suspicion of a gastrinoma [9].

Glucagonomas, caused by the autonomous hypersecretion of glucagon, are present in less than 3% of MEN1 patients, though NF-PanNETs may also show positive immunostaining for glucagon. These tumors arise from the alpha cells of the pancreas, more frequently occur in the pancreatic tail, and can be metastatic at the time of diagnosis [39,40]. Patients often present with a characteristic skin rash (necrolytic migratory erythema), weight loss, anemia, stomatitis cheilosis, deep vein thrombosis, and diabetes mellitus [39]. 

VIPomas are characterized by increased plasma levels of VIP and the clinical syndrome of watery diarrhea, hypokalemia, and achlorhydria [6,39,41]. 

Somatostatinomas are caused by autonomous hypersecretion of somatostatin. They are very rare tumors, with an incidence of 1 in 40 million, and almost 7% are associated with MEN1 [42]. Clinical presentation includes hyperglycemia, weight loss, anemia, and diarrhea, as well as cholelithiasis and obstructive symptoms due to their large size [43]. Somatostatinomas are mostly found within the pancreatic head and they have a high metastatic potential [6,39,44].

### 4.1. Genetics

Since the identification of the MEN1 gene, at least 1698 mutations, either germline or somatic, have been documented [45]. NETs in MEN1 patients arise due to a combination of genetic and epigenetic changes, including loss of heterozygosity (LOH) at 11q13 locus, the precise site of the MEN1 gene, leading to the biallelic loss of MEN1 and gene promoter hypermethylation [16,31]. 

PanNETs in MEN1 are clinically and genetically heterogeneous [45]. MEN1-associated PanNETs have undergone evaluation regarding their telomere length. Telomeres are specific chromatin formations that safeguard the ends of chromosomes. The alternative lengthening of telomeres (ALT) is a telomerase-independent mechanism that cancer cells activate to hinder telomere reduction, thereby enabling regular proliferation of somatic cells [31]. MEN1-associated PanNETs that are ALT positive are associated with a high rate of disease relapse. Additionally, a recent study investigated the role of ATRX (alpha thalassemia/mental retardation X-linked) and DAXX (death domain-associated protein) tumor suppressor genes’ protein expression by immunohistochemistry and telomere status by telomere-specific fluorescence in situ hybridization in 109 PanNETs from 28 MEN1 patients; ATRX and/or DAXX expression was lost in 6% of PanNETs. All these tumors were ≥3 cm in size, and showed alternative lengthening of telomere phenotype. Lymph node metastases were found in two out of the three tumors, and each metastasis had identical alterations in relation to the main tumor. Thus, these findings suggested that loss of ATRX and/or DAXX expression correlated with the alternative lengthening of telomere phenotype, greater tumor size of PanNETs and increased metastatic potential [46,47,48].

Epigenetic factors, particularly microRNAs (miRNAs), have also been implicated in the tumorigenesis in MEN1 [31]. It has been suggested that epigenetic mechanisms triggered by environmental factors may influence the disease phenotype in patients carrying the same MEN1 mutation [45,49]. The lack of a direct genotype–phenotype correlation in MEN1 tumorigenesis may be attributed to epigenetic factors acting as cofactors to genetic mutations [50]. Altered epigenetic regulation of gene expression has been suggested as a potential candidate for novel therapeutic strategies in MEN1 tumorigenesis [7,13]. Furthermore, menin has been found to epigenetically repress Hedgehog signaling in MEN1 patients, highlighting its potential role as a target for treating MEN1 tumors [51]. Moreover, MEN1 has been associated with altered epigenetic reprogramming, resulting in alpha-cell to beta-cell transdifferentiation [52]. 

### 4.2. Diagnosis and Surveillance

The high prevalence and malignant potential of NF-PanNETs outline the importance of early diagnosis and intervention. A systematic review showed that tumor markers, like chromogranin A, pancreatic polypeptide (PP), or glucagon, are not helpful in diagnosing NF-PanNETs in MEN1 patients due to their low sensitivity and specificity [53]. Hence, patients with MEN1 should perform regular imaging follow-up to identify the presence of a PanNET at an early stage. The most commonly used radiological modalities are magnetic resonance imaging (MRI), computed tomography (CT), and EUS. It has been shown that CT has lower sensitivity for detecting MEN1-related PanNETs compared to EUS [53]. Furthermore, cumulative exposure to ionizing radiation makes CT less useful as a radiologic long-term screening modality. MRI has been found to be more sensitive than CT and has been suggested as the method of choice [7,54]. Hyperintense and hypervascular tumors on T2-weighted images raise suspicion of a PanNET. However, concerns have been raised because of the accumulated gadolinium and its effects on the nervous system [55]. A systematic review concluded that EUS is the most sensitive method for identifying NF-PanNETs but it is invasive, operator-dependent, and has a lower sensitivity for PanNETs located at the pancreatic tail [53]. In addition, the vast majority of MEN1-related PanNETs is grade 1 and histological examination is not routinely recommended [56]. However, EUS allows histopathological confirmation of pancreatic lesions in rare cases of ambiguous imaging findings or for evaluating the grade of PanNETs that grow faster than expected [57]. 

It has been suggested that in order to ensure maximum sensitivity, both MRI and EUS could be used alternately to detect lesions as early as possible [54,58]. Consensus for optimizing personalized diagnostic and follow-up strategies is still needed, but MRI, due to its availability and noninvasive nature is considered the modality of choice [7,59]. The recent advances in somatostatin receptor (SSTR) positron emission tomography (PET)-CT (SSTR-PET-CT) in sporadic PanNETs have also been applied in MEN1 patients [60]. A study evaluating the role of SSTR-PET-CT in MEN1 patients showed that it can detect up to three times more PanNETs than conventional imaging [61]. SSTR-PET-CT has been suggested especially as a surveillance supplementary imaging modality for growing PanNETs > 10 mm and for the detection of occult metastasis before further therapeutic interventions are considered [6,53,59].

Current guidelines recommend initiating radiological screening for PanNETs at the age of 10 years, while a recent study suggested to postpone imaging until the age of 16 years in the absence of signs and symptoms [16,62]. A recently published study from the DMSG database showed that only 5 of 350 individuals who were followed for 9 years developed clinically relevant NF-PanNETs (size ≥ 20 mm or showing rapid growth) before the age of 18 years, of whom only 2 developed lymph node metastases [29]. Furthermore, it was observed that the estimated ages at which there was a 1%, 2.5%, and 5% risk of having a clinically relevant PanNET were 9.5, 13.5, and 17.8 years, respectively [29]. Hence, the authors suggested to commence the surveillance of MEN1 patients for PanNETs at age 13 to 14 years.

It is currently suggested to perform annual screening for insulinoma by fasting glucose starting at the age of 5 years and for gastrinoma by fasting serum gastrin (FSG) levels starting at the age 20 years [16]. However, others suggest to postpone biochemical and radiological screening in asymptomatic patients at least until the age of 16 years [6]. Routine biochemical testing, mainly glucose levels, and imaging intervals can be extended from 1 to 2–3 years in asymptomatic patients [6].

Similarly to NF-PanNETs, imaging evaluation of functioning NETs includes conventional imaging (CT and/or MRI), EUS, and SSTR-PET-CT [9]. However, the localization of insulinoma among other PanNETs may often be difficult due to the concomitant presence of NF-PanNETs, making the decision on the type and extent of surgery complex [63]. Glucagon-like peptide-1 receptors (GLP-1R) are overexpressed in 93% of localized insulinomas [64]. Therefore, GLP-1R PET/CT may be a helpful tool in differentiating insulinomas from other PanNETs present in MEN1 patients and guiding successful surgical intervention, albeit it is does not seem to be useful in metastatic insulinomas [6,9].

### 4.3. Management

The management of asymptomatic NF-PanNETs is evolving. Several studies have shown that most MEN1-related NF-PanNETs have an indolent course and grow slowly, with tumor growth ranging from 0.1 to 1.32 mm per year [33,34]. A recent study that evaluated the growth rate of PanNETs with EUS showed that PanNETs with size of <10 mm remained stable, whereas those with a diameter of >10 mm grew with a median rate of 0.44 mm/year [65]. It has previously been suggested that the conservative management for patients with NF-PanNETs < 2 cm was associated with a low risk of disease-specific mortality [66]. A recent study assessing the metastatic potential of dpNETs in MEN1 patients showed that tumors of 2 cm in size or more were associated with an increased risk of metastases [67]. Thus, the European Neuroendocrine Tumor Society (ENETS) suggests conservative follow-up for NF-PanNETs ≤ 2 cm, while surgical resection is advised for NF-PanNETs > 2 cm [30]. The DMSG suggests that surgical resection should be considered for NF-PanNETs ≥ 2 cm, those with increasing tumor size, as well as grade 2 (Ki67 > 3%) tumors or in the presence of suspicious lymph nodes [68,69]. There are still controversies regarding the extent of surgery as oncological resection should counterbalance the preservation of patients’ quality of life and the prevention of further progression or dissemination of the disease [6,30]. Therefore, it has been suggested that a minimally invasive approach, optimally with a laparoscopic pancreatic resection, may be considered [70].

The timing and indication of surgery for MEN1-related gastrinomas remain controversial as an excellent prognosis has been observed in non-operated patients with 10-year survival rates of 54% in cases with disseminated distant metastases, while acid-related complications may be effectively treated with PPIs [7,71]. In addition, most MEN1-associated gastrinomas are located in the duodenum [9,72,73]. The most commonly suggested surgical approach includes an exploration of the duodenum via duodenotomy or even resection of the duodenum, enucleation of pancreatic head lesions, and systematic lymphadenectomy with or without distal pancreatectomy according to the presence of other PanNETs [9]. Surgical resection is suggested for patients with tumor size larger than 2 cm or in cases where rapid tumor progression over a period of 6 to 12 months occurs [74,75,76]. Surgery may be considered in MEN1-associated metastatic gastrinomas confined to the liver if at least 90% of the identifiable tumor burden can safely be removed [6]. In cases of concomitant pHPT, parathyroidectomy precedes treatment for gastrinoma as elevated calcium levels stimulate gastrin secretion [9].

Surgical resection is recommended in case of localized insulinomas. Since the risk of nodal metastases is low, parenchyma-sparing pancreatic resection, including enucleation and central pancreatectomy, is considered as the first-line surgical strategy [9]. EUS-guided radiofrequency ablation (EUS-RFA) is a novel technique that has been shown to be effective in treating localized insulinomas and may be an alternative option in patients that are not fit for surgery [77,78].

A recent study that prospectively evaluated the effectiveness of long-acting somatostatin analogue (SSA) Lanreotide in patients with MEN1-related PanNETs < 2 cm showed that Lanreotide was associated with a significantly longer progression-free survival (PFS) compared to active surveillance (median progression time not reached vs. 40 months, *p* < 0.001) [79]. In addition, studies in cell lines and animal models have shown that MEN1 mutations lead to an upregulation of the enzyme dihydroorotate dehydrogenase (DHODH), and administration of the DHODH inhibitor, leflunomide, attenuates cell growth and tumor progression [80]. Preliminary studies in three MEN1 patients with PanNETs have reported promising results, but further investigation is required to support its use in MEN1-associated PanNETs [80].

The management of advanced metastatic MEN1 PanNETs is similar to sporadic tumors and requires a combination of surgical, interventional, hormonal, antiproliferative, and supportive measures and should be discussed within a multidisciplinary team [9,30]. SSAs, sunitinib, everolimus, peptide receptor radionuclide therapy (PRRT), and chemotherapy may be recommended according to tumor grade, extent of disease, growth rate, and patients’ performance status.

### 4.4. Prognosis

Patients with MEN1 have a decreased life expectancy compared to the general population, with TNETs and dpNETs being the most important disease-related cause of death [17,28]. As the sensitivity of modern imaging modalities has been progressively increasing, smaller PanNETs may be recognized, and the stratification of these tumors according to risk of aggressiveness and development of metastases remains a challenge [7,69]. Subgroups of small NF-PanNETs displaying faster growth and metastatic spread are identified and the early, reliable prognostic estimation may guide the proper management and surveillance [7]. 

A recent systematic review found that the most important prognostic factors in MEN1 related NF-PanNETs were the tumor size and grade [69]. NF-PanNETs of size ≥ 2 cm or grade 2 tumors should be considered at higher risk for metastases. Furthermore, significant tumor growth during follow-up has been also identified as a further risk factor [6]. Data from the DSMG database showed that surgery for NF-PanNETs was not associated with a significantly lower risk of liver metastases or death in NF-PanNETs < 2 cm in size, and such tumors can be managed by watchful waiting without loss of oncological safety [81]. A GTE and AFCE (Association Francophone de Chirurgie Endocrinienne) cohort study including 603 patients with MEN1-related dpNETs reported that the presence of ZES, tumor size > 2 cm, and age > 40 years were independently associated with an increased risk of metastases [67]. 

Gastrinomas are associated with an increased risk of distant metastases irrespective of tumor size, but this does not seem to be associated with significant impact on survival [7,28,66]. Results from the DMSG showed that MEN1-related gastrinomas are associated with decreased life expectancy, and FSG levels ≥ 20× upper limit of normal, size of PanNETs ≥ 2 cm, and the presence of liver metastases are independent prognostic factors of overall survival (OS) [82]. The 5- and 10-year OS rates for patients with MEN1-related gastrinoma were 83% and 65%, respectively, significantly lower compared to MEN1 patients without gastrinoma [82].

On the contrary, patients with insulinoma have a good prognosis with lower risk of metastases while they have also a lower risk of death after developing metastases [35,67].

Several authors have focused on the phenotype–genotype correlations in MEN1 patients, reporting a more aggressive behavior associated with certain genotypes, but no consistent results have been observed [7,29,50]. In addition, a recent study found that distant metastases from MEN1-related PanNETs were associated with a higher cumulative methylation index [31]. However, further studies are required to validate these results and implement them in clinical practice.

A recent international study assessed the prognostic value of circulating polyamines evaluating a 3-marker plasma polyamine signature (3MP) in patients with MEN1 and metastatic dpNETs and in a mouse model. It was observed that 3MP was associated with 66.7% sensitivity at 95% specificity for distinguishing patients from controls in an independent test set, suggesting a probable role of plasma polyamines as a prognostic factor for MEN1-related dpNETs [83,84].

## 5. Lung NETs in MEN1

Although LNETs are relatively rare in patients with MEN1, their estimated prevalence is 2–8% and 3–30%, based on histological or radiological identification, respectively [10,11,13,85]. The GTE cohort study reported that 4.8% of MEN1 patients had histologically proven LNETs while the DMSG identified radiologically suspicious lung lesions in approximately 23% of an MEN1 cohort followed for a median of 6.6 years [11,12]. The median age of diagnosis is 43 years and no impact has been observed for smoking status or specific genotypes [12]. 

Most MEN1-related LNETs are well differentiated, with the majority being TC, and are associated with an indolent course and good prognosis [7,12]. The DMSG observed an annual increase in tumor diameter of approximately 6% and a tumor doubling time of close to 12 years, while tumor growth was not associated with genotype, gender, smoking status, age at diagnosis of the LNET, and baseline tumor size [12]. However, distant metastases were observed in 16% of the GTE cohort and 3% of the DMSG cohort patients [11,12]. In addition, GTE reported five patients with LCLC or SCLC, albeit the causal relationship with MEN1 was unclear due to the long-term follow-up, the high frequency of smokers, and lack of molecular analysis [7,11].

LNETs are in the majority of asymptomatic cases, and screening for these tumors is based on radiological assessment. Rare cases present with symptoms related to hormonal hypersecretion, including carcinoid syndrome, Cushing’s syndrome, and acromegaly [10]. Current guidelines suggest CT or MRI imaging for the early detection of bronchial lesions [16]. Taking into consideration the indolent course of these tumors and the potential harm of frequent chest imaging, the follow-up intervals could be longer but this approach could result in delayed diagnosis of an aggressive LNET. Thus, it is considered that LNET screening protocols should be discussed with the patient and further actions should be based on well-informed decision making [7,12]. 

Optimal management for MEN1-related LNETs is yet to be determined. Surgical resection is considered the first-line treatment in localized LNETs [10]. However, in the DMSG, no significant difference was observed in terms of survival between operated and nonoperated patients [12]. Hence, in the case of small LNETs without lymph node involvement, a watch-and-wait approach may be recommended to determine the growth rate [10,12]. Nonsurgical locoregional procedures or SSAs may be suggested instead of anatomic pulmonary resection in order to preserve lung function. However, in the case of clinical or radiological progression during follow-up, surgery should be performed [12]. In selected cases, sub-lobar surgical resections including wedge resection can be considered [10]. The management of advanced and metastatic disease is similar to sporadic cases [10].

The 10-year survival of MEN1 patients with LNETs is approximately 88%, and LNET diagnosis does not seem to decrease OS in MEN1 patients [12]. The DMSG did not identify any prognostic factors of survival, while in the GTE cohort patients with TC had better survival than patients with AC, as did patients without node involvement or distant metastases [11,12]. A recent study reported that patients with MEN1-related LNETs had significantly lower disease-specific mortality compared to sporadic LNETs, but further research is required to verify these findings [86].

TNETs are rare neoplasms that are often sporadic, but nearly 25% of TNETs can be associated with MEN1 [87]. Their prevalence is 2–8% among patients with MEN1 [7]. They usually occur around the age of 40–50 years and the youngest age that a TNET has been reported is 16 years [7,32]. TNETs can be functioning, leading to Cushing’s syndrome due to hypersecretion of Adrenocorticotropic Hormone (ACTH). The 5-year survival of the TNET patients is approximately 62.5% and the 10-year survival is 31.3% [87,88].

TNETs often follow an aggressive course and contribute significantly to the morbidity and mortality in MEN1 patients [10]. Resectability is the key factor in prognosis [87]. Prophylactic thymectomy during initial parathyroidectomy in MEN1, as well as prophylactic thymectomy in young MEN1 mutation carriers with a family history of TNET, has been suggested to reduce the risk [7,10]. Median sternotomy and thoracotomy with thymus resection and lymph node dissection for TNETs is recommended [10]. Specific guidelines for MEN1-related TNETs are not available, but a similar approach to AC is usually employed in the presence of metastatic disease [10,87]. European Society for Medical Oncology (ESMO) guidelines suggest that in patients with slowly progressing advanced Somatostatin Receptor Imaging (SRI)-positive TNET, SSAs along with locoregional therapy, including surgery, are advised as the first line of treatment [10]. PRRT (based on positive uptake at SRI) can be considered as an alternate second-line treatment in advanced TNETs that are morphologically progressive or have a significant tumor burden [10]. ESMO suggests Temozolomide with or without capecitabine as first-line, and platinum-based chemotherapy as second-line, options in patients with progressive advanced disease. Everolimus can also be considered as second-line treatment [10].

## 6. Von Hippel–Lindau Syndrome (VHL)

Von Hippel–Lindau (VHL) is an autosomal dominant syndrome due to mutations of the VHL gene, a tumor suppressor gene located at chromosome 3p25 that predisposes individuals to the development of multiple benign and malignant neoplasms. The annual incidence of VHL is approximately 1 in 36,000 live births [89]. VHL is related to tumors in several organs, including the central nervous system (CNS) and retinal hemangioblastomas, pheochromocytomas and paragangliomas, renal cell carcinomas (RCC), PanNETs, endolymphatic sac tumors and cysts in the kidneys, pancreas, liver, testicles, and broad ligament [90].

Normally, the VHL protein (pVHL) plays a key role in the ubiquitination of the α subunit of the hypoxia-inducible factor (HIFα) under normal oxygen levels, leading to its degradation. Under hypoxic conditions, HIFα constitutively accumulates and forms heterodimers with HIF1β, acting as transcription factors that drive cell proliferation, angiogenesis via Vascular Endothelial Growth Factor (VEGF) upregulation, and erythropoiesis by upregulating the erythropoietin encoding gene (Figure 1) [89]. In VHL disease, the low levels of pVHL lead to an unregulated accumulation of HIFα, in a state called pseudohypoxia, with subsequent upregulation of proangiogenic peptides and development of highly vascular neoplasms [90,91]. The diagnosis of VHL is based on clinical criteria, with or without the presence of a relevant hereditary history or the molecular diagnosis that can be established by the identification of a pathogenic variant of VHL gene [89,92]. The clinical diagnosis of VHL requires the presence of two hemangioblastomas or one hemangioblastoma and a visceral neoplasm based on the presence of at least two VHL-related manifestations [93]. The VHL disease shows high penetrance, with 90% of affected individuals displaying symptoms by the age of 65 years [2]. Four VHL phenotypes (type 1, type 2A, type 2B, and type 2C) have been suggested based on the presence of pheochromocytoma or RCC. VHL type 1 and type 2 are characterized by a low and high risk for a pheochromocytoma, respectively (Table 2) [14].

The prevalence of PanNETs in patients with VHL syndrome ranges between 11 and 17%, exhibiting a female predominance [6,89,94,95], whereas in 7.6% of patients with VHL syndrome, only the pancreas is affected [14]. The VHL-related PanNETs display distinct clinical characteristics compared to sporadic PanNETs. Patients with VHL syndrome commonly have multiple PanNETs that are often cystic and are almost exclusively nonfunctioning. The presence of PanNETs has been described from an early age, most commonly starting from the second decade of life [95]. VHL-syndrome-related PanNETs mostly follow an indolent course and have a low risk for developing metastases. Hence, they have a good prognosis, although a minority of patients may exhibit a more aggressive disease [74].

Similarly to sporadic PanNETs, the size of the pancreatic lesions is the main risk stratification factor [89]. Lesions with a diameter of <1.5 cm in size have a low risk for metastasis or tumor progression, while PanNETs > 3 cm are associated with an increased metastatic risk [95,96]. In addition, a high tumor growth rate, defined as tumor diameter doubling time <500 days, and missense VHL gene variants or variants in exon 3 are considered risk factors for metastatic disease [89,96].

Current recommendations suggest surgical resection for PanNETs with diameter 3 cm or larger in patients with VHL syndrome [89]. Pancreatic NETs between 2 to 3 cm in size located at the pancreatic head should also be considered for surgical resection. Enucleation or limited resection should be considered for VHL-syndrome-related lesions, considering the increased risk of recurrence and the potential compromise of pancreatic function due to the presence of cystic disease [89]. However, recently published results from a European–American–Asian–VHL–PanNET registry showed an increased risk of metastases in PanNETs with a diameter ≥ 2.8 cm and in patients with mutations in exon 3, especially in codons 161/167 [95]. Hence, the authors concluded that to improve the outcome and survival, patients with PanNETs ≥ 2.5 cm in diameter, regardless of location within the pancreas, are strong candidates for surgery.

In case of advanced VHL-syndrome-related PanNETs, systemic treatment according to the guidelines for sporadic NETs is recommended [89]. However, considering the pathogenic mechanism of tumorigenesis in VHL disease, HIF and VEGF inhibition are considered the main targets of medical treatment [90]. Tyrosine Kinase Inhibitors (TKIs) with VEGF receptor inhibition ability, including sunitinib, vantetanib, pazopanib, sorafenib, and axitinib, have been evaluated in patients with VHL disease [90]. The efficacy of pazopanib for the treatment of VHL-related RCC was assessed in a phase II trial including 32 patients. This cohort also included 9 patients with 17 pancreatic lesions, predominately serous cystadenomas, and 9 of them (53%) displayed a partial response to treatment with pazopanib [95,97].

Belzutifan, an HIF2α inhibitor, has been recently approved by the US Food and Drug Administration (FDA) for VHL-related tumors, including PanNETs [98]. A phase II clinical trial including 61 patients with VHL, showed an objective response rate of 49% and 30% for RCC and CNS hemangioblastomas [98]. Twenty-two patients had PanNETs, and among them, the overall response rate was 83%. A recently published case report showed a partial radiographic response after one month of treatment with belzutifan in a patient with a metastatic PanNET [99]. There is no extensive evidence regarding the efficacy of belzutifan in cases of advanced VHL-related PanNETs, but this agent currently represents the most promising therapeutic modality, and its use may be considered an additional option in the management of VHL patients.

## 7. Tuberous Sclerosis Complex (TSC)

Tuberous sclerosis complex (TSC) is an autosomal dominant disorder characterized by hamartomas in several organs, disabling neurologic disorders, and dermatologic features [100]. 

TSC is attributed to mutations in one of two genes, the tuberous sclerosis complex 1 gene (TSC1) or TSC2, encoding hamartin and tuberin, respectively [14]. The hamartin and tuberin form a dimer that inhibits the mTOR pathway that regulates tumor growth and proliferation. Central nervous system tumors and renal disease are the main cause of morbidity and mortality in patients with TSC. 

Functioning and nonfunctioning PanNETs have rarely been reported in individuals with TSC, including cases of insulinomas or gastrinomas, while malignant PanNETs have also been observed [2,14] (Table 3). Sixteen cases of nonfunctioning PanNETs were identified in a cohort of patients with TSC with a reported frequency of 0.65% [101]. Functioning PanNETs are identified early due to the presence of symptoms related to hormonal secretion, while nonfunctioning tumors may be missed during early surveillance. Special attention and fine pancreatic cuts are recommended during imaging surveillance. Biopsy is suggested in the case of large-sized tumors, showing suspicious characteristics or displaying rapid growth rate [102].

Functioning PanNETs require diagnostic evaluation and management, as in patients with sporadic tumors [102]. In a series of 10 patients with TSC operated on for nonfunctioning PanNETs, there was low risk of complications and recurrence [103]. In addition, a recently published case series provided preliminary evidence supporting the slower growth rate of TSC-associated PanNETs in patients taking an mTOR inhibitor compared to those with no treatment, but further investigation is required to suggest the use of mTOR inhibitors as the first-line medical treatment or an alternative to surgical removal in TSC patients with nonfunctioning PanNETs [101].

**Table 3:** Pancreatic neuroendocrine tumors (PanNETs) in tuberous sclerosis complex patients.

Sex	Type of PanNET	Age at NET Diagnosis (Years)	Reference
F	Insulinoma	24	Gutman & Leffkowitz (1959) [104]
M	Insulinoma	23	Davoren & Epstein (1992) [105]
M	Gastrinoma	34	Schwarzkopf & Pfisterer (1994) [106]
M	Insulinoma	28	Kim et al. (1995) [107]
F	Insulinoma	18	Boubaddi et al. (1997) [108]
M	Malignant islet cell tumor	12	Verhoef et al. (1999) [109]
M	Insulinoma	43	Eledrisi et al. (2002) [110]
M	Malignant islet cell tumor	6	Francalanci et al. (2003) [111]
M	Islet cell neoplasm	39	Merritt et al. (2006) [112]
M	Well-differentiated PanNET	15	Arva et al. (2012) [113]
F/M	Well-differentiated PanNET	12	Koc et al. (2017) [114]
M	Well-differentiated PanNET	10	Bombardieri et al. (2013) [115]
F	Well-differentiated PanNET	35	Mortaji et al. (2017) [116]
M	Well-differentiated PanNET	3.5	Mehta et al. (2019) [117]
9M–7F	16 cases of nonfunctioning PanNETs	15.5–25.5	Mowrey et al. (2021) [101]

F: female, M: male.

## 8. Neurofibromatosis Type 1 (NF1)

Neurofibromatosis type 1 (NF1) is an autosomal dominant condition associated with an estimated birth incidence of approximately 1 in 2500 [118]. NF1 is attributed to germline mutations in one of the two alleles of the tumor suppressor gene NF1 located on chromosome 17q11.2 and somatic loss of function in the second allele that results in tumor development. Approximately 42% of affected individuals appear to have de novo mutations. The NF1 gene product, neurofibromin, regulates the activation of Ras proto-oncogene and influences cell growth and differentiation [118]. 

The clinical manifestations of NF1 involve multiple organs and include café au-lait macules, cutaneous and plexiform neurofibromas, iris hamartomas, optic gliomas, and bone abnormalities [118]. The clinical picture of affected individuals varies significantly, even among members of the same family. The diagnosis of NF1 is based mainly on clinical criteria but genetic testing may be required in equivocal cases [119]. 

Mainly duodenal somatostatinomas have been reported in patients with NF1, but they are relatively rare [14]. They develop mostly in the peri-ampullary region and present frequently with symptoms and signs of biliary and pancreatic duct obstruction [14]. Although these tumors stain positive for somatostatin, they are almost always hormonally silent and do not cause the somatostatinoma syndrome. Regional lymph node or liver metastases are observed in 30% of cases [120]. In addition, some rare case reports of nonfunctioning PanNETs, gastrinomas, and insulinomas have also been described in patients with NF1 [2,121]. A case of a 52-year-old female patient with NF1 and the ZES syndrome with a metastatic pancreatic gastrinoma has recently been reported [121].

## 9. Multiple Endocrine Neoplasia Type 4

Multiple endocrine neoplasia 4 (MEN4) syndrome is caused by germline mutations in the cyclin-dependent kinase (CDK) inhibitor 1b gene (*CDKN1B*) which encodes for the p27^Kip1^, commonly referred to as 27 or KIP1 [5,122]. *CDKN1B* is a tumor suppressor gene that regulates cell cycle progression, and its mutations affect the cellular localization of p27, stability, or binding [5]. MEN4 has a prevalence of less than one per million, with varied penetrance and heterogeneous phenotypic expression that may include different manifestations, even among members of the same family [4,5]. Approximately 1.5–3.7% of patients with MEN1-like phenotype and negative genetic testing for MEN1 have *CDKN1B* mutations [122]. A recent systematic review reported that the majority of patients diagnosed with MEN4 are women, while the median age for the presentation of the first endocrine disorder is 43.5 years [122]. The most frequently affected tissues are the parathyroid glands (75%), mostly as a uniglandular disease, followed by the pituitary gland (44%), the pancreas (15%), the thyroid gland (8%), the adrenal gland (6%), and the thymus (4%). 

Four patients with NF-PanNETs (one metastatic, one nonmetastatic, and two multifocal nonmetastatic) and three patients with pancreatic gastrinomas (two metastatic) have been reported so far [122]. In addition, two patients with nonmetastatic gastric NETs and one with a metastatic small intestinal NET have been described [122]. In the DMSG cohort, three patients with LNETs were found to harbor *CDKN1B* mutations [12]. The management approach for MEN4-related NETs is similar to that of MEN1, but data regarding the characteristics and natural course of MEN4 associated tumors are still scarce.

### Future Directions

Recent studies have focused on identifying prognostic and predictive biomarkers, such as measuring polyamine levels, to identify “high-risk” individuals with dpNETs for developing future distant metastases. Additional markers could play a significant role in both diagnostic and therapeutic strategies [82,83]. Moreover, studies have assessed the plasmatic circulating cell-free DNA (ccfDNA) in patients with MEN1 or VHL [123,124]. A case study of a patient with MEN1 mosaicism showed that ccfDNA from thymic variants were detected a month before the relapse of TNET, showing that the TNET was growing, and ccfDNA was suggested as a potential early tumor marker [123]. In another case study with metastatic clear renal cell carcinoma, ccfDNA was investigated, and changes in variant allele frequency (VAF) of VHL mutation were associated with the tumor size assessed by radiographic images [124]. The NETest is a standardized liquid biopsy for NETs that assesses the expression of 51 NET genes using a real-time polymerase chain reaction. It offers an accurate molecular profile and has an additional predictive role in determining the clinical progression of NETs. Its role has not been studied yet in hereditary syndrome-related NETs but it could be considered as a predictive tool for the clinical course of these NETs in future studies [125,126]. However, a major unmet need is the identification of further, highly specific prognostic and predictive tools aiming to identify those PanNETs and LNETs with potentially aggressive behavior and guide towards an individualized surveillance and interventional process. Considering that such patients are not amenable to a complete cure, it is important to assess patients’ and families’ psychological burden. Several studies have evaluated health-related quality of life (HRQOL) particularly in MEN1 patients. A study has reported higher levels of anxiety, depression fatigue, pain interference, sleep disturbance, and diminished physical and social functioning in MEN1 patients compared to controls [126]. A further study showed no differences between patients with or without MEN1, while a Dutch study reported significant financial burden and unemployment in such patients that were correlated to worse HRQOL [126,127,128]. Incorporating HRQOL assessments into the monitoring program will offer a deeper understanding of the perceived disease burden experienced by patients with hereditary syndromes either at presentation, during the course of the disease, or during disease progression. This will ultimately enhance the quality of care for these people. Collaborative management of individuals with MEN1 by a multidisciplinary team, ideally with the inclusion of a psychologist, is of outmost importance. 

## 10. Conclusions

Although the vast majority of PanNETs and LNETs are sporadic, a significant proportion are associated with hereditary syndromes, including MEN1, MEN4, VHL, NF1, and TSC. These syndromes display a high penetrance, while clinical manifestations may vary significantly, even among members of the same family. PanNETs associated with hereditary syndromes are often multiple and develop at an earlier age compared to sporadic tumors. LNETs are relatively rare and develop mainly in the context of MEN1, leading to a more aggressive course in the presence of atypical histology. Although the majority of PanNETs and LNETs follow a relatively indolent course in addition to being malignant, in some cases they can become aggressive and contribute to disease-related morbidity and mortality. In the last decades, more information has been gained regarding the natural course of NETs associated with inherited syndromes, and the patients’ outcomes have significantly improved. However, a major unmet need is the identification of further prognostic and predicting tools aiming to identify those PanNETs and LNETs with potentially aggressive behavior and guide towards an individualized surveillance and interventional approach. Patients with hereditary syndromes should be treated in specialized centers in the context of a multidisciplinary team that ensures adequate management as well as promotes data collection and initiates international research collaborations in order to further improve patients’ outcome.

## Figures and Tables

**Table 1:** Clinical phenotype of hereditary syndromes associated with PanNETs and LNETs.

Syndrome	Type of NET	Other Malignant Tumors	Benign Manifestations	Frequency
Multiple endocrine neoplasia type 1 (MEN1)	PanNET Duodenal NET LNET TNET Gastric NET	Adrenocortical cancer Breast cancer	Primary hyperparathyroidism Pituitary adenoma Adrenal adenoma Angiofibroma Collagenoma Meningioma Lipoma	Prevalence 1–10 in 100,000
von Hippel–Lindau (VHL)	PanNET	Clear cell renal carcinoma Pheochromocytoma Endolymphatic sac tumor	Hemangioblastoma Renal cyst Pancreatic serous cystadenoma Epididymis cystadenoma	1 in 36,000
Tuberous sclerosis complex (TSC)	PanNET	Renal-cell carcinoma Astrocytoma Angiomyolipoma	Angiofibroma Shagreen patch Fibroma Retinal hamartoma Ash leaf macules	1 in 12,000–14,000
Neurofibromatosis type 1 (NF1)	PanNET Duodenal NET	Gastrointestinal Stromal Tumor (GIST) Optic glioma Meningioma Astrocytoma Neurofibrosarcoma Pheochromocytoma Breast cancer	Café au lait macule Neurofibroma Axillary/inguinal freckling Lisch nodules Bone abnormalities	Prevalence 1 in 3000
Multiple endocrine neoplasia type 4 (MEN4)	PanNET Duodenal NET LNET Gastric NET Small intestinal NET		Primary hyperparathyroidism Pituitary adenoma	Prevalence 1 in 1,000,000

**Table 2:** VHL clinical phenotypes.

Type 1	Renal cell carcinoma (RCC), retinal hemangioblastoma, CNS hemangioblastoma, low risk for a pheochromocytoma
Type 2	Retinal hemangioblastoma, CNS hemangioblastoma, high risk for a pheochromocytoma
Type 2A	Low risk for RCC
Type 2B	High risk for RCC
Type 2C	Risk for pheochromocytoma only

## Data Availability

The data presented in this study are available in this article.

## Abbreviations

ENETS	European Neuroendocrine Tumor Society
EURACAN	European Reference Network on Rare Adult Cancers
